# Sex-differential testosterone response to long-term weight loss

**DOI:** 10.1038/s41366-024-01591-7

**Published:** 2024-07-16

**Authors:** Malgorzata M. Brzozowska, Dana Bliuc, Artur Mazur, Paul A. Baldock, John A. Eisman, Jerry R. Greenfield, Jacqueline R. Center

**Affiliations:** 1 Garvan institute of Medical Research, Darlinghurst, NSW Australia; 2 Sutherland and St George Hospitals, Caringbah, NSW Australia; 3 University of New South Wales Sydney, Faculty of Medicine, Sydney, NSW Australia; 4 University of Rzeszow, Faculty of Medicine, Rzeszow, Poland; 5 University of Notre Dame Australia, School of Medicine Sydney, Sydney, NSW Australia; 6 St Vincent’s Hospital Clinical School, Department of Endocrinology, Darlinghurst, NSW Australia

**Keywords:** Obesity, Preclinical research

## Abstract

**Objectives:**

Obesity-associated gonadal dysfunction is a common comorbidity in patients seeking weight loss interventions. We examined the incremental effect of weight loss on gonadal axes in men and women over 3 years. Changes in sex hormones were compared between dietary intervention (Diet) and bariatric procedures: Roux-en-Y gastric bypass (RYGB), sleeve gastrectomy (SG) and laparoscopic adjustable gastric banding (LAGB). Additional analysis assessed changes in corticotropic, somatotropic and thyroid axes after weight loss interventions.

**Methods:**

This prospective, observational study included 61 adults with Body Mass Index >30 kg/m^2^, mean age 51 (SD = 11) years. Endocrine parameters were measured at baseline and at 6 timepoints over 36-months.

**Results:**

For each 1 kg of weight lost, between baseline and 36 months, total testosterone increased by 0.6% (95% CI: 0.2%, 1.0%, *p* = 0.002) in males and decreased by 0.8% (95% CI: −1.4%, −0.3%, *p* = 0.003) in females. These changes remained statistically significant when controlled for age and for menopausal status in females. At 36 months, in comparison with Diet, RYGB women had lower total testosterone by 54% (95% CI: −90%, −17%, *p* = 0.004), reduced free androgen index (FAI) by 65% (95% CI; −114%, −17%, *p* = 0.009) while SG had reduced FAI by 39% (95% CI; −77%, 0%, *p* = 0.05). No such differences between groups were noted for male subjects. Adrenocorticotropic hormone declined by 0.3% (95% CI: 0.0, −0.5%, *p* = 0.05), insulin-like growth factor-1 increased by 0.4% (95% CI; 0.2%, 0.7%, *p* = 0.005), without such thyrotrophin change for each 1 kg of weight loss, for entire cohort, over 36 months.

**Conclusions:**

The testosterone changes observed in this study were proportional to the amount of weight loss. In females, reduction in androgens was independent of age and menopausal status and more pronounced after bariatric procedures. This study finding warrants further clinical research to explore an impact of androgen reduction on functional and cognitive status in postmenopausal women. The observed changes in pituitary hormones may contribute to the metabolic benefits of bariatric surgery.

## Introduction

Obesity, an increasingly common medical condition, is associated with chronic low-grade inflammation with altered adipocytokines, proinflammatory cytokines and hypothalamic hormones, which may affect the gonadal function through a complex set of mechanisms [1, 2]. Notably, in previous studies, obesity-associated gonadal dysfunction was highlighted as a common comorbidity in patients seeking weight loss.

The majority of studies focusing on female gonadal dysfunction exclusively included premenopausal women and focused on women affected by the common female endocrinopathy polycystic ovary syndrome (PCOS) [3, 4]. The literature consistently highlights that bariatric surgery alleviates clinical and biochemical hyperandrogenism in PCOS women with reduction in total and free testosterone levels, with subsequent restoration of regular menstrual pattern and improved fertility [5, 6].

The cross-sectional studies consistently highlight lower testosterone levels in males with obesity with declining testosterone levels in longitudinal studies [7]. In men, excess body fat is associated with lower testosterone levels due to the conversion of testosterone into oestrogen in fat tissue [8]. A reduction in sex hormone-binding globulin (SHBG), which mediates androgen delivery to the peripheral tissue, fosters the reduction in total testosterone [9]. Insulin resistance can further decrease testosterone levels with prevalence of hypogonadism in up to 50% in men with type 2 diabetes mellitus [1]. Finally, deficiencies in nutrients like zinc and vitamin D may contribute to lower testosterone levels [10].

The negative energy balance associated with weight loss interventions influences the activity of hypothalamo-pituitary- adrenal -thyroid, -and -somatotropic axes and therefore regulation of cortisol, duration of stress responses and growth hormone release [11–17].

We have previously demonstrated that bariatric surgery is a more effective long-term weight loss modality than conventional medical therapy in alleviating obesity related inflammation and insulin resistance [18, 19]. Based on these findings, we hypothesised that effective weight loss interventions will lead to the several alterations in the hypothalamic-pituitary hormones affected by obesity and that these hormonal changes, will be more pronounced in the surgical groups due to their more marked weight loss.

The primary aim of this study was to assess the incremental effect of weight loss on endogenous sex hormones including testosterone, oestradiol (E2) and SHBG in men and women over the period of 3 years. The secondary aim was to examine the long-term effect of three different types of bariatric surgery Roux-en-Y gastric bypass (RYGB), sleeve gastrectomy (SG) and laparoscopic gastric banding (LAGB) in comparison with dietary intervention (Diet) on changes in gonadal axes. We also explored the effects of weight loss on corticotropic (assessed by adrenocorticotropic hormone (ACTH) and cortisol), somatotropic (assessed by insulin-like growth factors (IGF1) pathway)) and thyroid axes (thyroid stimulating hormone (TSH), free thyroxine (FT4) and free triiodothyronine (fT3)) over the period of 3 years.

## Methods

### Study design, setting and study participants

This was a prospective and observational study of adult participants with obesity, aged between 18 and 70 years, who underwent 3 types of bariatric surgery and dietary intervention and were followed for 3 years.

The study protocol was previously published [18]. Inclusion criteria were body mass index (BMI) ≥ 30 kg/m^2^ and presence of obesity for at least 5 years, despite attempts to lose weight through other measures [19]. Exclusion criteria were pregnancy or planning a pregnancy within 2 years, being within 5 years post onset of menopause or presence of an active psychiatric problem that would limit adherence to the study protocol [18].

The study was designed to enrol 60 study participants aiming for 15 study subjects in each study group. Eligible participants were recruited from Obesity clinics at Royal Prince Alfred and Royal North Shore Hospitals and from private bariatric services at St George Private and St Vincent’s Hospitals in Sydney, between years 2009 and 2012 and then followed until October 2015. The study participants were allocated to either their bariatric or dietary interventions based on their probability of diabetes remission criteria, which were congruent with DiaRem score [20]. Sixty-four participants were recruited, of whom 3 withdrew over the time of the study. The remaining 61 subjects were included in the analysis.

**Fig. 1:**
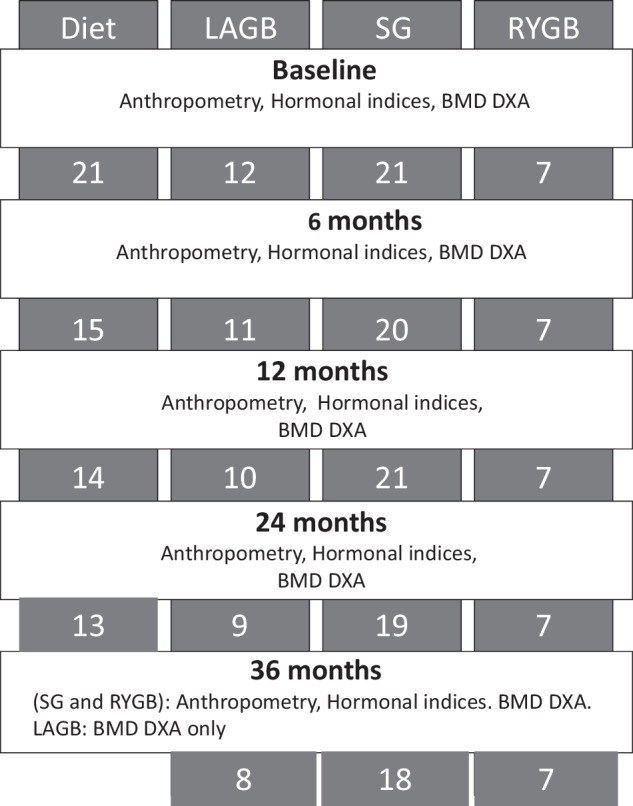
Flow of study participants and study procedures. Each panel displays the number of study participants recorded at each study timepoint. BMD DXA scan Bone Mineral Density scan, LAGB laparoscopic gastric banding, SG sleeve gastrectomy, RYGB Roux-en-Y gastric bypass.

Anthropometric and hormonal parameters were recorded at baseline and at 1-, 3-, 6-, 12-, 24- for all participants and 36-months (for SG and RYGB) post interventions, see Fig. 1. The weight changes of study subjects were previously reported and as expected, surgical groups lost significantly more weight than the Diet group [18].

### Ethics approval and consent to participate

Ethical approval was obtained from the St Vincent’s Hospital Health Research Ethics Committee, Sydney HREC/09/SVH/64. Informed written consent was obtained from all participants. All methods were performed in accordance with the Declaration of Helsinki and in compliance with the National Health and Medical Research Council in Australia (NHMRC) National Statement on Ethical Conduct in Human Research (NHMRC, 2007). The study was listed in Australian New Zealand Clinical Trials Registry (ANZCTR), 12613000188730.

## Analytical methods

### Primary outcome measurement

Blood samples were obtained after 12 h overnight fasting at baseline and at each study visit. Serum samples were measured for total testosterone, SHBG (CV < 4%), and oestradiol by electro chemiluminescent immunoassay (Elecsys kit; Roche Diagnostics).

The free androgen index (FAI) was used to estimate physiologically active testosterone. This index was calculated in females as the ratio of total testosterone divided by SHBG (both expressed in the same units) and multiplied by 100 to yield numerical results comparable in free testosterone concentration. As FAI tends to overestimate calculated free testosterone (CFT) in males the Vermeulen equation was used to calculate CFT in male participants [21].

### Assessment of corticotropic, somatotropic and thyroid axes

Serum cortisol and ACTH were measured by Roche Modular E170 immunoassay with CV < 6% at all levels tested. IGF1 was measured using ROCHE Elecsys immunoassay with established sex-and age-based reference intervals. The TSH assays were determined by Roche Modular analyser.

The detailed description of analytical methods is included in Supplementary online document.

### Statistical analyses

Baseline characteristics of study subjects were compared between surgical groups vs dietary group. Normally distributed variables were presented as mean (±SD) and non-normally distributed variables were presented as median (±interquartile range). Analysis of variance (ANOVA) and post-hoc pairwise Tukey honest significance difference test, or Kruskal–Wallis’ test and post-hoc pairwise Dunn’s test examined the comparisons between Diet and surgical groups for normally or non-normally distributed baseline data, respectively. The study had over 90% power to detect the observed differences between RYGB and diet groups with a type 1 error of 5%.

#### Primary study aim

As there was no interaction between study procedures and study visits for all assessed hormonal variables, the differences in these parameters were examined for the whole cohort of participants at each timepoint in comparison with baseline values. The changes in the gonadal axes were examined separately for male and female subjects. Differences in testosterone, SHBG, E2, LH, FSH, pre-and post-weight loss at baseline, 6, 12, 24 and 36 months were assessed by Wilcoxon sign rank test.

Random intercept linear mixed effects model assessed the contribution of weight loss (exposure) to changes in individual sex hormone (outcome variable) over 36 months.

The covariates were age and in the female group, menopausal status. The hormonal variables were log transformed. Effects of weight loss on sex hormone changes were examined in unadjusted models followed by an adjusted model by age and, in women, menopausal status.

#### Secondary study aim

The contribution of each bariatric surgery vs Diet to changes in hormonal indices over 3 years was assessed using individual random intercept linear mixed effects model for each individual hormone and expressed as difference in mean percentage changes over time between specific bariatric surgery and Diet (95% confidence interval: corresponding *p* value).

Exploratory analyses using Wilcoxon sign rank test (ACTH, cortisol and IGF1) and paired t-test (TSH, FT4 and FT3) examined longitudinal changes in these parameters for the whole cohort of subjects while random intercept linear mixed effects model assessed the contribution of weight loss and bariatric procedures to changes in individual hormones.

## Results

### Baseline characteristics (see Table 1)

There were 61 study participants with RYGB (*N* = 7), SG (*N* = 21), LAGB (*N* = 12) and Diet-treated (*N* = 21) subjects followed over 36 months. There were no baseline differences between surgical groups and Diet in sex, testosterone, SHBG, E2, Luteinizing hormone (LH), Follicle-stimulating hormone (FSH), IGF1, ACTH and cortisol. Patients in the SG group were heavier in comparison with Diet. The women in LAGB group were younger with median age of 42.5 years (IQR: 32.9,55.7) than Diet group by 10.2 years (95% CI: −14.4, −6.0, *P* < 0.0001). Thirty-one percent of women in the study were premenopausal. There were baseline differences in TSH (*P* = 0.01). The TSH value, in comparison with Diet, was lower in GS group by 1.47 mIU/L (95% CI: −2.48, −0.47, *p* = 0.004) and in RYGB by 1.50 mIU/L (95% CI: −2.81, −0.18, *P* = 0.026).

**Table 1:** Baseline characteristics of patient cohort.

Parameter (reference range)	Diet *N* = 21	LAGB *N* = 12	SG *N* = 21	RYGB *N* = 7	Total *N* = 61	*P* value
All participants						
Weight (kg)*	110.9 (26.0)	105.0 (16.5)	125.3 (16.9)	113.1 (15.9)	115 (22.6)	**0.04**
BMI, kg/m^2*.^	38.1 (6.6)	37.7 (4.5)	42.5 (5.3)	42.3 (7.7)	40.2 (6.7)	0.06
Age (years)*	53.4 (8.6)	42.2 (13.0)	50.0 (11.7)	50.9 (7.2)	50.9 (11.4)	0.07
Cortisol nmol/L, (200–600)	270 (220, 327)	284 (249, 306)	298(203, 368)	274 (243, 340)	280 (216, 351)	0.65
ACTH pmol/L, (0–12)	4.3 (2.7, 5.9)	4.4 (3.5, 4.62)	5.2 (2.4, 6.7)	4.1 (3.6, 5.4)	4.4 (3.2, 6.2)	0.44
IGF1 nmol/L, (RR 10–30)	13.1 (10.9, 18.0)	17.9 (15.2, 19.7)	12.8 (10.5, 17.6)	14 (13.8, 16.5)	14.9 (11.3, 18.7)	0.41
FT4 pmol/L, (RR 11–22)	14.4 (13.8, 16.5)	15.3 (14.2, 17.4)	15.4 (13.9, 16.5)	15.6 (14.2, 17.6)	15.2 (13.8, 17)	0.82
FT3 pmol/L, (RR 3–6.2)	4.5 (4.3, 4.9)	4.9 (4.5, 5.3)	5 (4.65, 5.3)	4.7 (4.35, 5.1)	4.8 (4.3, 5.3)	0.08
TSH mIU/L, (RR 0.4–4.2)	2.1(1.3, 3.2)	1.36 (1.25, 2.3)	1.5 (1.1, 2.4)	1.7 (1.4, 2.9)	1.6 (1.2, 2.6)	**0.01**
Females						
Sex (Females), *N* (%)	17 (81%)	10 (83%)	12 (57%)	5 (71%)	44 (72%)	0.11
FSH (IU/L), Postmenopausal Females: (25–140)	19.7 (5.6, 55)	9.2 (5.6, 36.4)	28.8 (4.7, 44.7)	36.2 (34.7, 58.1)	27.4 (5.7, 53.6)	0.90
LH (IU/L), Postmenopausal Females: (7.7–60)	15.2 (6.9, 29.2)	11.5 (5.2, 28.5)	14 (5.7, 28.0)	28 (21.7, 29)	17.3 (5.7, 30.0)	0.92
Oestradiol pmol/L Postmenopausal Females: <100	103 (46, 294)	127 (84.2, 234)	74 (59.5, 91)	72 (49, 76)	87 (54.2, 218)	0.72
SHBG nmol/L, (20–110)	34.3 (21.9, 48.9)	30.0 (24.4, 46.2)	25.6 (19.6, 106)	24.7 (20, 27.1)	29.1 (21.3, 49)	0.39
Testosterone nmol/L, (0.0–1.8)	0.8 (0.6, 1.25)	1.15 (0.6, 1.45)	1.15 (0.75, 1.65)	0.7 (0.5, 0.8)	0.9 (0.6, 1.4)	0.71
Males						
Sexr (Males), *N* (%)	4 (19%)	2 (17%)	9 (43%)	2 (29%)	17 (28%)	
FSH (IU/L), Males (1.5–13)	6.45 (5.6, 7.4)	4.7 (4.5, 4.95)	3.7 (3.0, 4.5)	4.7 (4.7, 4.7)	4.2 (3.7, 6.3)	0.32
LH (IU/L), Males: (1.7–8.6)	5.4 (4.9, 6.55)	2.65 (2.6, 2.7)	3.7 (2.5, 4.4)	5.6 (5.6, 5.6)	4.0 (2.7, 5.3)	0.12
Oestradiol pmol/L, (0–160)	85 (76.5, 93.5)	126 (125, 128)	93 (78, 132)	162 (162, 162)	113 (80.5, 131)	0.27
SHBG nmol/L, (20–90)	20.1 (17.9, 24)	25.5 (21.2, 29.8)	24.5 (16.6, 30.8)	17 (17, 17)	20.1 (17, 31.6)	0.56
Testosterone nmol/L, (12–36)	13.7 (9.95, 17.2)	18.2 (15.0, 21.5)	11.8 (9.5, 13.7)	11.7 (11.7, 11.7)	11.8 (10.1, 16.4)	0.91

Parameters values are expressed as median (interquartile range-IQR) unless specified, (*) - mean (SD), *P* value for comparison of surgical procedures with Diet group.

*ACTH* adrenocorticotropic hormone, *BMI* body mass index, *FSH* Follicle-stimulating hormone, *IGF1* insulin-like growth factor, *LH* Luteinizing hormone, *SHBG* sex hormone-binding globulin, *TSH* thyroid stimulating hormone, *FT4* free thyroxine, *FT3* free triiodothyronine, *LAGB* laparoscopic gastric banding, *SG* sleeve gastrectomy, *RYGB* Roux-en-Y gastric bypass.

Bolded values indicate statistical significance for comparison of surgical procedures with Diet group.

A single male SG patient was affected by hypogonadotropic hypogonadism with a baseline total testosterone of 4.2 nmol/L (RR 12–36). None of female participants had biochemical hyperandrogenism. The cohort of premenopausal women had total testosterone of 0.9 nmol/L (IQR: 0.6, 1.62), SHBG of 36.8 nmol/L (IQR: 24.0, 52.8), FSH of 5.2 IU/L (IQR: 4.1, 8.98), LH of 5.7 IU/L (IQR: 4.35, 9.15) and oestradiol of 187 pmol/L (IQR: 97, 368). The postmenopausal women had total testosterone of 0.9 nmol/L (IQR: 0.6, 1.3), SHBG of 27.5 nmol/L (IQR: 20.2, 43), FSH of 47.9 IU/L (IQR: 28.5, 63.6), LH of 26.7 IU/L (IQR: 17.3, 31.9) with oestradiol of 70 pmol/L (IQR:48.2, 99).

None of the study subjects had evidence of adrenal insufficiency or glucocorticoid excess. Eight of sixty-one (13%) patients had reduced IGF1 level prior to their weight loss intervention.

### Change in weight over time

Over the 3 year study period, when % of total weight change was analysed, in comparison with baseline weight, women on average lost: RYGB −38% (95% CI: −48%, −29%), %), SG −22% (95% CI: −30%, −14%), LAGB −17% (95% CI: −30%, −4%), Diet −2% (95% CI: −7%, 4%) of weight while males experienced following weight change RYGB −21% (95% CI: −29%, −14%), SG −22% (95% CI: −27%, −17%), LAGB −16% (95% CI: −28%, −4%), Diet 4% (−25%, 33%).

### Female participants

1. The association between weight loss and sex hormones over time.
  - Absolute changes over time for the whole cohort of female patients (see Table 2):

**Table 2:** Median (IQR) values for sex hormones at each time point.

Parameter IQR (Q1, Q3)	Baseline	6 months	12 months	24 months	36 months
Females					
Testosterone, nmol/L, (0.0–1.8)	0.9 (0.6, 1.4)	**0.6 (0.2, 0.9)**	**0.6 (0.5, 0.9)**	**0.6 (0.2, 0.85)**	0.7 (0.2, 0.95)
SHBG, nmol/L (20–110)	29.1 (21.3, 49)	**47.8 (29.5, 70.1)**	**47.0 (33.6, 64.6)**	**56 (31.6, 75.5)**	**46.5 (39.5, 75.2)**
LH (IU/L), Postmenopausal Females: (7.7–60)	17.3 (5.7, 30.0)	15.8 (4.75, 34.6)	22.5 (6.6, 37.6)	**28.4 (7, 41.9)**	25.6 (13.6, 36.0)
FSH (IU/L), Postmenopausal Females: (25–140)	27.4 (5.7, 53.6)	**33.1 (4.4, 65.4)**	**48.0 (9.85, 66.1)**	**52.1 (12.9, 71.4)**	**55.2 (37.0, 68.0)**
Oestradiol pmol/L, Postmenopausal Females: <100	87 (54.2, 218)	67 (49, 244)	53 (49, 172)	54.5 (49, 109)	49 (49, 56.8)
Males					
Testosterone, nmol/L, (12–36)	11.8 (10.1, 16.4)	15.7 (12.5, 20.0)	**17 (13.7, 19.8)**	**17.7 (14.3, 22.5)**	**17.8 (14.4, 19.4)**
SHBG nmol/L, Males (20–90)	20.1 (17, 31.6)	36.8 (32.1, 41.0)	**35.2 (28.3, 43)**	**41 (30, 50)**	37.5 (30.5, 43.5)
LH (IU/L), (1.7–8.6)	4.0 (2.7, 5.3)	4.9 (4.15, 5.65)	4.25 (3.08, 4.25)	**5.15 (4.3, 6.1)**	4.85(3.97, 5.6)
FSH (IU/L), Males (1.5–13)	4.2 (3.7, 6.3)	4.9 (4.0, 6.8)	4.45 (3.4, 5.2)	4.65 (3.8, 5.6)	5.1 (4.2, 9.3)
Oestradiol pmol/L, (0–160)	113 (80.5, 131)	103 (87.8, 112)	104 (72.2, 130)	106 (88, 120)	102 (69.8, 130)

Bolded values indicate statistical significance for changes in parameters at each timepoint in comparison with the baseline values.

*FSH* Follicle-stimulating hormone, *LH* Luteinizing hormone, *SHBG* sex hormone-binding globulin.
    Total testosterone value declined from baseline values 0.9 nmol/L (IQR: 0.6, 1.4) to 0.6 nmol/L (IQR: 0.2, 0.9), *P* < 0.0001 at 6 months and these changes were maintained at 12 and 24 months. The SHBG increased from 29.1 nmol/L (IQR: 21.7, 49.3) to 47.8 nmol/L (IQR: 29.5, 70.1, *P* < 0.0001) at 6 months and remained raised up to 36 months at 46.5 nmol/L (IQR: 39.5, 75.2, *P* = 0.0053). There was a significant increase in baseline LH (at 24 months) and baseline FSH (up to 36 months) without change in oestradiol levels at any timepoint.
  - Univariate and multivariate results from mixed effect model (see Table 3):

**Table 3:** Unadjusted and multivariate analysis adjusted mean difference (95% CI) for sex hormones between baseline and 36 months per 1 kg weight loss.

Parameter	Univariate analysis	Multivariate analysis^a^
	Trend estimate (kg/36 months; 95% CI)	
Females		
Testosterone	**−0.8% (−1.4% to −0.3%)**	**−0.9% (−1.4% to −0.3%)**
SHBG	0.5% (0.0% to 0.9%)	0.4% (−0.1% to 0.9%)
LH	0.8% (−0.1% to 1.6%)	−0.1% (−0.7% to 0.6%)
FSH	**1.3% (0.3% to 2.3%)**	0.1% (−0,6% to 0.7%)
Oestradiol	0.2% (−0.5% to 0.9%)	0.4% (−0.2 to 0.1%)
Males		
Testosterone	**0.6% (0.2% to 1.0%)**	**0.7% (0.3% to 1.1%)**
SHBG	**0.4% (0.0% to 0.8%)**	0.4% (0.0% to 0.7%)
CFT	**0.5% (0.0 to 0.9%)**	**0.7% (0.3% to 1.1%)**.
LH	0.2% (−0.2% to 0.6%)	0.1%(−0.5% to 0.3%)
FSH	0.5% (0.% to 1.0%)	**0.6% (0.1% to 1.1%)**
Oestradiol	0.1% (−0.3% to 0.5%)	0.2% (−0.1% to 0.6%)
All participants		
ACTH	0.2% (−0.1% to 0.4%)	**−0.3% (0% to −0.5%)**
Cortisol	0.1% (−0.2% to 0.3%)	0.1% (−0.1% to 0.3%)
IGF1	**0.4% (0.1% to 0.6%)**	**0.4% (0.2% to 0.7%)**
TSH	0% (−0.4% to 0.5)	0.1% (−0.4% to 0.6%)
FT4	**−0.2% (0 to −0.3%)**	**−0.2% (0% to −0.3%)**
FT3	**−0.1% (0% to −0.2%)**	**−0.1% (0% to −0.2%)**

Bolded values indicate statistical significance.

*ACTH* adrenocorticotropic hormone, *CFT* calculated free testosterone, *FSH* Follicle-stimulating hormone, *IGF1* insulin-like growth factor, *LH* Luteinizing hormone, *SHBG* sex hormone-binding globulin, *TSH* thyroid stimulating hormone, *FT4* free thyroxine, *FT3* free triiodothyronine.

^a^Adjustment for the participants age (all patients) and menopausal status (females).
    There was a significant positive relationship between weight loss and serum testosterone.
    Over 36 months for each kg of weight loss total testosterone decreased by 0.8% (−1.4%, −0.3%), independently from their age and menopausal status.
    After adjustment for lost weight SHBG increased by 10% at each visit (95% CI: 4.7%, 14.7%, *P* < 0.0001). FSH rise was inversely associated with weight loss, no longer significant after controlling for subjects’ age and menopausal status.
2. The impact of bariatric surgery type vs diet on sex hormones changes over time (see Table 4):

**Table 4:** Changes (%) in gonadal axis between baseline and 3 years in comparison with Diet group.

	Unadjusted mean difference (%) in hormonal indices from Diet group (95% CI)		
Parameter	LAGB	SG	RYGB
Female participants			
Testosterone	−2% (−34% to 30%)	18% (−11% to 46%)	**−54% (−90% to −17%)**
SHBG	7% (−20% to 34%)	**51% (27% to 76%)**	12% (−19% to 44%)
FAI	−9% (−52% to 33%)	**−39% (−77% to 0%)**	**−65% (−114% to −17%)**
LH	−**53% (−101% to −5%)**	−28% (−73% to 15%)	42% (−13% to 97%)
FSH	**−71% (−128% to −15%)**	−41% (−93% to 10%)	39% (−26% to 103%)
Oestradiol	**49% (11% to 88%)**	−3% (−38% to 33%)	−27% (−70% to 18%)
Male participants			
Testosterone	37% (−7% to 79&)	−0.1% (−26% to 26%)	17% (−24% to 57%)
SHBG	14% (−32% to 60%)	13% (−14% to 40%)	−19% (−62% to 25%)
CFT	35% (−10% to 81%)	−5% (−33% to 23%)	33% (−11% to 76%)
LH	**−51% (−90% to −13%)**	−21% (−45% to 3%)	1% (−36% to 37%)
FSH	−28% (−82% to 27%)	**−39% (−73% to −5%)**	12% (−64% to 40%)
Oestradiol	16% (−16% to 61%)	−7% (−33% to 19%)	22% (−16% to 61%)

Significant changes in examined parameters are highlighted in bold.

*CFT* calculated free testosterone, *FAI* free androgen index, *FSH* Follicle-stimulating hormone, *LH* Luteinizing hormone, *SHBG* sex hormone-binding globulin levels, *LAGB* laparoscopic gastric banding, *RYGB* Roux-en-Y gastric bypass, *SG* sleeve gastrectomy.

The women post RYGB procedure achieved on average lower testosterone by 54% (95% CI: −90%, −17%, *P* = 0.004) and lower FAI by 65% (95% CI; −114%, −17%) than Diet and this difference remained significant by 43% (95% CI; −5.4%, −81%) after controlling for participants’ age and weight changes. The FAI was lower by 39% (95% CI; −77%, 0%) in SG group without such differences in testosterone or FAI between Diet and LAGB group. Only women post SG had average higher SHBG by 51% (95% CI: 27%, 76%, *P* = 0.0001) than Diet with significant difference by 50% (95% CI: 25%, 75%) after controlling for their age and weight.The younger LAGB group achieved on average lower FSH (71%; 95% CI: −128%, −15%, *P* = 0.014) and lower LH (53%; 95% CI: −101%, −5%, *P* = 0.032) and higher oestradiol (49%: 95% CI: 11%, 88%, *P* = 0.012) than Diet however these values were no longer different to Diet after controlling for age and lost weight. No such changes were observed for any other surgical groups.

## Male participants

1. The association between weight loss and sex hormones over time.
  - Absolute changes over time for the whole cohort of male patients (see Table 2):
    Total testosterone values increased from baseline 11.8 nmol/L (IQR: 10.1, 16.4) up to 17.8 nmol/L (IQR: 14.4, 19.4, *P* = 0.035) at 36 months with SHBG rise from baseline 20.1 nmol/L (IQR: 17, 31.6) to 41 nmol/L (IQR: 30, 50, *P* = 0.034) at 24 months.
    No change from baseline in FSH or E2 was noted at any of the study timepoints, however the LH increased from 4.0 IU/L (2.7, 5.3) to 5.15 IU/L (4.3, 6.1) at 24 months.
  - Univariate and multivariate results from mixed effect model (see Table 3):
    There was an inverse association between lost weight and serum testosterone level. For each 1 kg weight reduction male subjects’ testosterone level increased by 0.6% (95% CI: 0.2%, 1.0%), after controlling for participants’ age by 0.7% (0.3%, 1.1%), the calculated free testosterone increased by 0.5% (95% CI: 0.0, 0.9%, *P* = 0.042).
    The SHBG increased by each study visit by 6.1% (95% CI: 1.8%, 11%, *P* = 0.013). For each 1 kg of weight loss, the SHBG level increased by 0.4% (95% CI: 0.0, 0.8%, *P* = 0.033) with no longer significant difference after adjustment for participants’ age.
    An inverse relationship was noted between lost weight and FSH (after controlling for participants’ age) without such association for LH or oestradiol.
2. Effect of each bariatric surgery vs diet on sex hormones changes over time (see Table 4):

There were no differences in testosterone and SHBG between Diet and surgical groups. In comparison with Diet FSH declined in GS group by 39% (95% CI: −73%, −5%, *P* = 0.024), not significantly different after adjustment for weight and age. The LH declined in LAGB procedure by 51% (95% CI: −90%, −13%, *P* = 0.010) and remained significantly different by 52% (95% CI: 9%, 97%, *P* = 0.019) after adjustment for their weight and age. No differences were noted in E2 between study groups.

### Exploratory analysis of changes in corticotropic, somatotropic and thyroid axes over 36 months

When compared with their baseline values ACTH levels decreased significantly by 12 months, IGF1 values increased significantly by 24 months and TSH levels declined by 36 months for entire cohort of patients, (Supplementary Table 1). Weight loss was associated with decline in ACTH and with IGF1 increase without change in TSH. For each 1 kg of lost weight, adjusted for participants’ age, the ACTH level declined by 0.3% (95% CI: 0.0, −0.5%, *P* = 0.050) while IGF1 increased by 0.4% (0.2%, 0.7%), FT4 declined by 0.2% (0, −0.3%) and FT3 declined by 0.1% (0%, −0.2%) see Table 3.

All bariatric surgeries produced significant hormonal change compared to Diet: (RYGB for reduced ACTH by 22% (95% CI: −42%, −1%, *P* = 0.032), SG for increased cortisol level by 13% (95% CI: 0.1%, 25%, *P* = 0.048) and reduction in TSH by 54% (95% CI: −81%, −27%, *P* < 0.001), which remained significant after adjustment for age, LAGB for increased IGF1 by 26% (95% CI: 9%, 44%, *P* = 0.003), (Supplementary Table 2).

## Discussion

In the present study of 61 participants with severe obesity, we found that the changes in endogenous sex hormones in both women and men were proportional to their weight loss. The sustained weight loss was positively associated with reduction in total testosterone in females and inversely associated with increase in total testosterone and CFT in males. These androgen changes were more pronounced for female participants who underwent bariatric procedures without such differences between medical and surgical weight loss interventions for male subjects.

In contrast to our study, the majority of bariatric studies exclusively included premenopausal women and primarily focused on those affected by polycystic ovary syndrome. Thus, there is a paucity of data related to changes in androgen levels in postmenopausal women with severe obesity undergoing weight loss interventions. In particular, limited studies examined the effects of bariatric surgery on circulating androgen levels that were within the normal range preoperatively.

Congruent with other report, the present study observed a marked reduction in androgens in women after their surgically induced weight loss, irrespectively of their menopausal state and preoperative testosterone levels [22]. In comparison with Diet group, RYGB procedure produced lower total testosterone while SG group achieved lower free testosterone and higher SHBG levels. Interestingly low levels of testosterone and DHEAS have previously been implicated in an increased risk for cognitive dysfunction, reduced mood, sexual drive, and frailty, particularly in older women [23, 24]. The long-term consequences of the reduction in normal values of androgen levels in older women with obesity remain to be explored.

Interestingly, our younger LAGB female group experienced some recovery of their gonadal axis with an increase in oestradiol and reduction in FSH and LH over time. This observation contrasts with results from meta-analysis which found that bariatric surgery led to raised LH and FSH in females after follow-up period longer than 12 months [6].

In approximately 40% of men, obesity is associated with low testosterone together with low SHGB and increased oestradiol, and with low or inappropriately normal gonadotropins [25]. In our study, despite an average age of male subjects of 55 years, only a single male was affected by hypogonadism. However, the median testosterone level of male subjects was below the reference range.

The weight loss interventions led to an increase in total testosterone over 12, 24 and 36 months with an increase in SHBG from baseline up to 24 months. The sustained increase in SHBG levels and in total and CFT in males was previously attributed to reduction in visceral adipose tissue and to alterations in testosterone metabolism with relative decreases in aromatization and lower 5α-reductase activity [1, 8, 26]. The restoration of testosterone levels may help to relieve fatigue and improve reduced libido [27]. As testosterone inhibits stem cell differentiation into adipocytes and promotes myogenesis, testosterone supplementation in males results in favourable change in body composition with reduction in total and abdominal fat mass and preservation of lean body mass [28, 29]. Notably recent recommendations by the American Association of Clinical Endocrinologists (AACE) endorsed screening men with obesity for hypogonadism and evaluation of body weight in men with hypogonadism [30].

The majority of studies which examined changes in gonadal axis induced by weight loss have been of short duration with younger age male participants [31]. Previous randomized controlled trial of male participants with type 2 diabetes reported improvements in total and free testosterone levels up to 5 years after bariatric surgery without significant changes in oestradiol levels [32]. The present study also observed that increase in testosterone was sustained over long term, suggesting that weight loss interventions had lasting effects on hormone balance. However, we did not find differences in androgens between bariatric and dietary interventions, likely due to the small sample size of males in our bariatric groups.

Our study findings, showing an inverse association between bariatric weight loss and an increase in testosterone levels in males, are supported by other studies reporting weight loss with testosterone therapy. A recent observational registry study evaluating the effects of testosterone therapy in men with testosterone deficiency reported that exogenous testosterone treatment contributed to significant long-term weight loss in hypogonadal men, comparable to the weight loss outcomes after bariatric surgery. In this trial, the percentage of total weight loss achieved was 20% in men with a BMI over 35 kg/m², 10% in men in the overweight category, and 5% in men with a normal BMI [33]. In hypogonadal men receiving long-term testosterone therapy, a significant reduction in BMI and waist circumference is linked to the restoration of steady and physiologically normal levels of total and calculated free testosterone with sustained therapeutic effects [34].

Additionally, we examined the weight loss driven neuroendocrine response with respect to hypothalamic-pituitary-adrenal, somatropic and thyroid axes. When all patients were analysed together their ACTH values declined below baseline at 12-months with improved GH secretion at 24 months post interventions. Contrary to previous studies reporting raised serum cortisol levels up to 6 months after bariatric surgery, we have observed stable serum cortisol level over the study period [35]. We observed no association between the magnitude of lost weight and between changes in cortisol secretion or TSH values. However, some differences were noted in these hormonal parameters between SG surgery and dietary interventions.

The study results highlighted a differential effect of weight loss interventions on the HPA axis with lower ACTH response in RYGB group in comparison with dietary intervention, which may impact on the long-term weight loss maintenance. Above results may reflect reduced activation of HPA axis, by bariatric procedure such as RYGB surgery, to an acute stress due to caloric restriction. A previous murine study observed, that despite equivalent weight loss, there were different responses of HPA axis to dietary and surgical weight loss interventions [36]. This body weight defence (obesogenic) mechanism is likely being reduced after some types of bariatric surgery, with RYGB procedure being a more effective long term weight loss modality [19, 36]. Further studies are needed to elucidate the contribution of HPA axis activity in humans to the homoeostatic regulatory mechanism responsible for weight loss after dietary and bariatric interventions.

Research on the effects of bariatric surgery on Growth Hormone (GH) levels, although limited, suggests a negative relationship between adiposity and GH values in patients with obesity. Congruent with our results reporting substantial number of patients with reduced IGF1 values, previous studies noted both normal and decreased IGF-1 level in patients with obesity [37–40]. These derangements of somatotropic axis, reflected by low IGF-1 levels, are linked with insulin resistance, type 2 diabetes and cardiovascular disease [41, 42].

In the present study the rise in IGF1 levels correlated with a greater weight loss. The LAGB group achieved higher IGF1 values in comparison with the Diet over 36 months. Notably some studies have reported a restoration of somatotropic axis alterations after bariatric surgery [43]. These data support the view that alterations of GH/IGF-1 axis in obesity may be reversible as successful weight loss may promote normal spontaneous and stimulated GH release [44, 45].

Significant weight reduction may also affect thyroid function, which plays a crucial role in regulating metabolism and energy expenditure. Congruent with results of previous metanalysis, we observed a decline in thyrotropin (TSH) and FT3 values but not in FT4 over 36 months [16, 46]. The TSH reduction was more pronounced following bariatric surgery than after dietary intervention, however, in contrast with some research TSH reduction did not correlate with the magnitude of lost weight. This suggests that regardless of weight loss, bariatric surgery may affect TSH hormone release, through additional pathways with a proposed mechanism of altered leptin secretion affecting pituitary-thyroid axis [47].

In summary, we have shown that weight loss, likely through multiple metabolic pathways, has effects on neuroendocrine regulation of pituitary axis. These changes can indirectly influence sex hormone levels with more prominent effects in surgical groups in comparison with dietary interventions. The circulating androgen levels markedly decreased in women with obesity independently of their pre or postmenopausal state as well in women with preoperatively normal androgenic levels. Interestingly, we have also noted a recovery of gonadal axis in the group of younger women who underwent LAGB surgery. The testosterone level increased in male participants and this change was inversely associated with the magnitude of weight loss.

Importantly there was a significant increase in SHBG in male and female participants, likely due to their changes in weight and body composition. As increases in SHBG levels lead to changes in the biologically active form of testosterone (free testosterone), these changes have implications for sexual health and fertility in patients undergoing bariatric procedures.

We have observed that weight loss interventions had positive effects on other aspects of neuroendocrine axis reducing hyperactivity of ACTH; limiting the stress response to the weight loss hence possibly reducing the subsequent weight regain in study participants. Additional effects on increase in IGF1 with weight loss, more prominent after LAGB procedure, and reduction in TSH in bariatric patients have likely contributed to improved insulin resistance, metabolic rate and improved body composition parameters in patients undergoing weight loss interventions.

### Strengths and limitations

The strengths of this study are its duration and prospective study design which allowed for examination of postsurgical hormonal changes over the extended time period (0–36 months). The adjustment for baseline characteristics of study participants, their age and weight changes and study procedures has allowed us to investigate the changes in hormonal indices above those associated with surgical procedures and the sustained weight loss [19]. Furthermore, the study analysis accounted for the baseline measurements and hierarchical clustered nature of the longitudinal data with repeated study measurements over the period of 3 years [19].

Our study is not free of limitations including relatively small sample size of study participants. We did not have data if improvement in their gonadal hormones resulted in improved personal and psychological health or sexual function. In the present study the participating women were not evaluated for the presence of a PCOS or irregularities of their menstrual cycles. Therefore, the clinical significance of these reductions in testosterone levels which were within the normal reference range is unknown. Furthermore, the limitations of this study include lack of randomization of participants to treatments such that observed differences between groups may be subject to residual confounding.

Overall, however our data support the notion that weight loss is of major value towards neuroendocrine health. Future research is needed to gain a deeper understanding of the specific mechanisms underlying the relationship between bariatric surgery and the neuroendocrine axis. Importantly individual responses to surgery can vary, as the long-term impact on the neuroendocrine axis may depend on several factors such as the type of surgery performed, patients’ pre-existing metabolic conditions, and their overall health status. Ongoing monitoring of hormone levels and metabolic parameters is crucial for patients undergoing bariatric procedures to ensure long-term optimal health outcomes.

## Supplementary information

Supplementary online Document.

## Data Availability

All data generated or analysed during this study are included in this published article (and its supplementary information files).
