# Correction: Sex-differential testosterone response to long-term weight loss

**DOI:** 10.1038/s41366-024-01597-1

**Published:** 2024-08-05

**Authors:** Malgorzata M. Brzozowska, Dana Bliuc, Artur Mazur, Paul A. Baldock, John A. Eisman, Jerry R. Greenfield, Jacqueline R. Center

**Affiliations:** 1 Garvan institute of Medical Research, Darlinghurst, NSW Australia; 2 Sutherland and St George Hospitals, Caringbah, NSW Australia; 3 University of New South Wales Sydney, Faculty of Medicine, Sydney, NSW Australia; 4 University of Rzeszow, Faculty of Medicine, Rzeszow, Poland; 5 University of Notre Dame Australia, School of Medicine Sydney, Sydney, NSW Australia; 6 St Vincent’s Hospital Clinical School, Department of Endocrinology, Darlinghurst, NSW Australia

**Keywords:** Obesity, Preclinical research

Correction to: *International Journal of Obesity* 10.1038/s41366-024-01591-7, published online 16 July 2024

In the original version of the article, the middle name initial of the author ‘Jerry R. Greenfield’ was missing. The original article has been corrected.

