# Supplementary material for: Sex-differential testosterone response to long-term weight loss
Source: Int J Obes (Lond). 2024 Jul 16;48(10):1481–8. doi: 10.1038/s41366-024-01591-7 (PMC11420080; doi:10.1038/s41366-024-01591-7)
Supplement: Supplementary file 1 — Supplementary online Document. [file 41366_2024_1591_MOESM1_ESM.pdf]

Supplementary online Document.

1) Analytical methods
2) Supplementary Table 1.
3) Supplementary Table 2.

1) Analytical methods:

1.1 Reference ranges for examined parameters:

a) Testosterone male reference ranges(RR) were as following:
- between 18 – 50 years: 10 nmol/L – 32 nmol/L,
- > 50 years: between 8 nmol/L – 32 nmol/L

b) Testosterone female reference ranges were as following:
- > 10 years: between 0 nmol/L – 1.8 nmol/L.

c) SHBG:
- Male (15 - 50 years): 15 – 50, (> 50 years): 20 – 90,
- Female (15 years and older): 20 – 110.

d) Female free androgen index (FAI) (15 years and older): 0 - 4.0.

e) Oestradiol reference ranges:
- postmenopausal females: < 100 pmol/L,
- males: 100pmol/L – 160 pmol/L

f) TSH (RR 0.4 to 4.2 IU/L), free T4 (RR 11.0 – 22 pmol/L), free T3 (RR 3.0 – 6.2 pmol/L);

g) Follicle-stimulating hormone (FSH): Female: Follicular: (RR 3.5 – 13 IU/L), Mid cycle: (RR 4.7 – 22 IU/L), Luteal: (RR 1.7 – 7.7 IU/L), Post menopausal: (RR 25.0 – 140 IU/L), Male:(RR 1.5 – 13 IU/L),

h) Luteinizing hormone (LH) Female: Follicular: (RR 2.4 – 13 IU/L), Mid cycle: (RR 14 – 97 IU/L), Luteal: (RR 1.0 – 12 IU/L), Post menopausal: (RR7.7 – 60 IU/L), Male: (RR 1.7 – 8.6 IU/L)

i) Morning cortisol samples (measured between 8 am and 10 am) (RR: 200 – 700 nmol/L), ACTH (RR 0-12 pmol/L).

1.2 Hormonal Assays:

IGF1 was measured using ROCHE Elecsys immunoassay with established sex-and age-based reference intervals, Cortisol was measured by Roche Modular E170 immunoassay, CV < 6% at all levels tested. Roche Modular analyser: TSH, free T4, free T3 , FSH, LH.

2) Supplementary Table 1. Median (IQR) values for examined hormones at each time point for all study participants.

Parameter IQR (Q1, Q3)	Baseline	6 months	12 months	24 months	36 months
ACTH pmol/L (0-12)	4.4 (3.2 , 6.2)	**3.8 (2.65, 5.05)**	**3.9 (2.9, 5.4)**	3.65 (2.95, 4.3)	4 (3.4, 5.5)
Cortisol nmol/L, (200-600)	280 (216, 351)	279 (203, 343)	306 (240, 380)	286 (227, 355)	314 (262, 368)
IGF1 nmol/L (RR 10-30)	14.9 (11.3, 18.7)	**16.2 (11.1, 20.6)**	15.9 (9.7, 20)	**16.5 (13.1, 21.5)**	14.3 (11.0, 18.9)
TSH mIU/L (RR 0.4-4.2)	1.6 (1.2, 2.6)	1.37 (0.9, 1.8)	1.46 (1.0, 2.2)	1.29 (1.0, 2.2)	**1.26 (0.97, 1.95)**
FT4 pmol/L (RR 11-22)	15.2 (13.8, 16.9)	15.8 (14.2, 17.3)	15.4 (14.0, 16.6)	15.6 (13.7, 16.6)	15.5 (13.8, 18.1)
FT3 mIU/L (RR 0.4-4.2)	4.8 (4.35, 5.3)	4.6 (4.3, 5.1)	**4.6 (4.23, 4.9)**	**4.6 (4.3, 4.9)**	4.65 (4.23, 4.8)

List of abbreviations: ACTH - adrenocorticotropic hormone, IGF1 - insulin-like growth factor, TSH - thyroid stimulating hormone, FT4 - free thyroxine, FT3 - free triiodothyronine.

Bolded values indicate statistical significance for changes in parameters at each timepoint in comparison with the baseline values.

3) Supplementary Table 2. Changes in examined hormones between baseline and 3 years in comparison with Diet group.

	Unadjusted mean difference (%) in hormonal indices from Diet group (95%CI)		
Parameter	LAGB	SG	RYGB
ACTH	-4% (-23% to 14%)	50% (-10% to 20%)	**-22% (-42% to -1%)**
Cortisol	3% (-12% to 18%)	**13% (95% CI: 0.1% to 25%)**	5% (-12% to 22%)
IGF1	**26% (9% to 44%)**	-6% (-20% to 9%)	5% (-15% to 25%)
TSH	30% (-62% to 3%)	**-54% (-81% to -27%)**	23% (-58% to 12%)

List of abbreviations: ACTH - adrenocorticotropic hormone, IGF1 - insulin-like growth factor, TSH - thyroid stimulating hormone, LAGB - laparoscopic gastric banding, SG- sleeve gastrectomy, RYGB-Roux-en-Y gastric bypass; P value for comparison of surgical procedures with Diet group.

Bolded values indicate statistical significance.
